# Interpretable machine learning models for predicting in-hospital and 30 days adverse events in acute coronary syndrome patients in Kuwait

**DOI:** 10.1038/s41598-024-51604-8

**Published:** 2024-01-12

**Authors:** Moh A. Alkhamis, Mohammad Al Jarallah, Sreeja Attur, Mohammad Zubaid

**Affiliations:** 1 Department of Epidemiology and Biostatistics, Health Sciences Center, College of Public Health, Kuwait University, Kuwait City, Kuwait; 2 Department of Cardiology, Sabah Al Ahmed Cardiac Center, Ministry of Health, Kuwait City, Kuwait; 3 Department of Medicine, Health Sciences Center, Faculty of Medicine, Kuwait University, Kuwait City, Kuwait

**Keywords:** Cardiology, Risk factors, Epidemiology, Computer science

## Abstract

The relationships between acute coronary syndromes (ACS) adverse events and the associated risk factors are typically complicated and nonlinear, which poses significant challenges to clinicians' attempts at risk stratification. Here, we aim to explore the implementation of modern risk stratification tools to untangle how these complex factors shape the risk of adverse events in patients with ACS. We used an interpretable multi-algorithm machine learning (ML) approach and clinical features to fit predictive models to 1,976 patients with ACS in Kuwait. We demonstrated that random forest (RF) and extreme gradient boosting (XGB) algorithms, remarkably outperform traditional logistic regression model (AUCs = 0.84 & 0.79 for RF and XGB, respectively). Our in-hospital adverse events model identified left ventricular ejection fraction as the most important predictor with the highest interaction strength with other factors. However, using the 30-days adverse events model, we found that performing an urgent coronary artery bypass graft was the most important predictor, with creatinine levels having the strongest overall interaction with other related factors. Our ML models not only untangled the non-linear relationships that shape the clinical epidemiology of ACS adverse events but also elucidated their risk in individual patients based on their unique features.

## Introduction

Cardiovascular diseases (CVD) continue to be the leading cause of unprecedented global public health and economic implications^1^. In fact, CVD mortalities represent approximately one-third of all deaths worldwide annually^2–4^. This is particularly true for Acute coronary syndromes (ACS), which constitute most of the mortalities caused by CVDs^3^. Cardiac catheterization, percutaneous coronary intervention (PCI), and angiography are considered the gold standards in ACS diagnosis and intervention among clinicians^5^. Among these tools, cardiac catheterization is the most common cardiac intervention approach used worldwide, with more than 1 million procedures being performed annually in the United States alone^6^. Yet, the complexity of the epidemiology of ACS poses a remarkable challenge to the intervention capacity of primary and secondary care clinicians, leading to patient-related and procedure-related complications. However, risk stratification tools developed in the past few decades remarkably helped clinicians improve their diagnostic and prognostic efforts. These risk stratification tools were derived from large population-based studies and traditional statistical methods designed to capture comparisons and insights into the risk factors that predict ACS adverse events resulting from patient clinical conditions and in-hospital procedures. Yet, most of these tools neglect complex interactions and non-linear relationships between such risk factors, which are the primary cause for hindering their prediction’s accuracy on the individual level and, hence, their epidemiological plausibility. Machine learning (ML) models do not assume linear relationships and flexibly accommodate higher-order interactions to make more robust individualized predictions^7,8^.

Accounting for interactions in clinical predictions is statistically and epidemiologically critical, as the range of complications is multifactorial, which, for example, depends on whether the procedure is diagnostic or interventional, patient demographics, comorbidities, clinical symptoms at the time of presentation, and experience of the operating clinician^6^. Also, complications can be either minor, such as discomfort at the operating site or significant such as myocardial infarction or death. Patients with severe comorbidities such as congestive heart failure or chronic renal failure are at higher risk of complications^9^. Furthermore, the long-term benefits and complications from highly invasive interventions combined with the patient's risk status, such as their age or comorbidities, need to be considered. Clinicians commonly resort to early risk stratification tools which are commonly used to classify patients’ susceptibility to different ACS events according to their individual risk profile at the time of presentation. Popular risk stratification includes thrombolysis in myocardial infarction (TIMI)^10^ or global registry of acute coronary events (GRACE)^11^, which provides an overall quantitative prognosis for patients with ACS. Further, most interventional cardiologists resort to the National Cardiovascular Data Registry (NCDR) for catheterization percutaneous coronary intervention (NCDR-CathPCI) contemporary mortality risk model to predict potential adverse events resulting from their intervention procedures^12^.

The intrinsic limitation of inferences derived from population-based data, such as registries or randomized clinical trials in terms of generalizability, may represent a critical challenge for improving cardiac intervention outcomes. For example, the emergency department's patient population or those excluded from the clinical trials might be underrepresented in such data^13,14^. Similarly, susceptible populations from certain geographical regions with specific genetic and environmental risk factors might also be unrepresented. Furthermore, in emergency or busy clinical settings, the early risk stratification of ACS patients may make intervention-related decisions fallible, leading to serious in or out-hospital implications^15^. Standard risk stratification tools can be used to customize personalized clinical interventions based on individual patient-specific predictions, and they mostly rely on a scoring system derived from a traditional statistical framework. However, these statistical frameworks mainly comprise stepwise regression models with many fixed assumptions on the nature of the data, including randomness, independence, and linear relationships between the risk factors and the outcome. These assumptions may make their generalizability to external cohorts unreliable in some circumstances, particularly when they require a preselected set of variables in the development stage, resulting in potential critical loss of information^16,17^.

Additionally, traditional regression models are highly susceptible to overfitting due to collinearity issues that arise between the risk factors when their dimension is significantly large (e.g., large population-based registry data)^18^. In contrast, ML models are remarkably flexible because they depend on minimal statistical assumptions and can robustly explore large volumes of data and a variety of variables (e.g., medical images and signals). Additionally, in the recent decade, a variety of modern statistical methods have been introduced that safeguard ML models from overfitting (e.g., k-fold cross-validation and feature selection)^19^. Therefore, they are more capable of capturing multidimensional non-linear complex relationships within clinical data and, thus, able to produce data-driven generalizable predictions^14,16,17^. Also, because ML-driven risk stratification approaches outperformed traditional scoring scales^7,8,14,16,17^, and the United States Food and Drug Administration has already approved the use of a few learning algorithms for intervention and diagnostic cardiology^20^. Here, we use a multi-algorithm ML ensemble statistical framework on a multicenter registry of ACS patients to investigate the factors that shaped the risk of in-hospital and 30 days adverse events. We used patient demographic, clinical characteristics, and clinicians’ intervention data to build two interpretable predictive risk models for short and long-term ACS adverse events. Moreover, we integrated and evaluated our models in the context of individual patient-level prognosis to address the advantages and limitations of our data-driven ML models in contrast to using traditional risk approaches in a clinical setting.

## Methods

### Data source

We retrieved our data from a prospective, multicenter, cohort-based registry, formally known as the Kuwait catheterization laboratory project (Kuwait CLAP) registry. The data comprises 1,976 records of all ACS patients undergoing coronary angiograms in two central and high-volume hospitals in Kuwait enrolled between February 16, 2020, and February 22, 2021. The first participating hospital has 500 beds and serves approximately 600,000 patients on an annual basis. At the same time the second hospital has 700 beds and serves approximately 1 million people. Patients were followed prospectively for the duration of their admission. A case report form was used to collect data that mainly included elements from the 2013 report of American College of Cardiology (i.e., ACCF/AHA key data elements and definitions for measuring the clinical management and outcomes of patients with acute coronary syndromes and coronary artery disease). The key data elements included definitions for measuring patients' clinical management and outcomes with acute coronary syndrome^21^. The form also had information about patient demographics, medical history, home medications, clinical management, in-hospital course, discharge, and 30-day follow-up data. The included patients ' follow-up data (mainly rehospitalization status) were collected via the hospital electronic records and telephone interviews after 30 days of discharge.

The Kuwait CLAP involving human subjects were reviewed and approved by each participating hospital's Ministry of Health central ethics committee and conformed to the ethical guidelines of the 1975 Declaration of Helsinki. In accordance with ethical guidelines for medical and health research involving human subjects in Kuwait, the requirement for written informed consent from the participants was waived by the review committee.

Here, we refer to our selected variables as ‘features’. We selected features thought to be relevant with a direct link to risk to the study outcomes. Thus, we reduced our data dimensionality to remove redundant information and improve computational efficiency, classification precision, data visualization and interpretation^22^. The final set of features included patient demographics, past medical history, presenting symptoms on admission, medication administered in the first 24 h from admission, in-hospital Cath-lab procedures, laboratory values before and after the In-hospital procedure, and discharge characteristics (Supplementary Table 1). We used in-hospital and 30 days of discharge adverse events as our study outcomes (Table 1). However, because many adverse events were rare and observed in the patients simultaneously (i.e., more than 85% of the affected patients had more than one adverse event), we aggregated them to compose our two defined outcomes. Thus, our first outcome is defined as patients who had one or more in-hospital adverse events post-catheterization/PCI, while the second outcome is defined as patients who were rehospitalized within 30 days after discharge due to an ACS event and/or related conditions (Table 1). Also, we used in-hospital adverse events as an independent predictor of adverse events 30 days after discharge. Additionally, discharge characteristics were excluded from the 30-day model (Supplementary Table 1). In this dataset, the aggregated prevalence of in-hospital and 30-day adverse events were 13.6% and 7.7%, respectively (Table 1).

**Table 1:** Summary profile of the observed adverse events in the patients’ cohort.

	*n_i_ (n_i_%)
Post CATH/PCI clinical events (*n = 269; 13.6%)	
Recurrent angina	59 (21.9%)
Infarction/reinfarction	19 (7.1%)
Cardiogenic shock	43 (16.0%)
Pericarditis	10 (3.7%)
Active endocarditis	1 (0.4%)
Contrast induced nephropathy	75 (27.9%)
Mechanical complications	4 (1.5%)
Temporary pacemaker	12 (4.5%)
Heart failure	86 (32.0%)
Cardiac arrest	24 (8.9%)
CVA/stroke	12 (4.5%)
Atrial fibrillation	42 (15.6%)
Ventricular tachycardia	28 (10.4%)
Mechanical ventilation	44 (16.4%)
New requirement for dialysis	11 (4.1%)
Bleeding	14 (5.2%)
RBC whole blood transfusion	12 (4.5%)
Acute stent thrombosis	12 (4.5%)
30 days outcomes after discharge/rehospitalization (n = 148; 7.7%)	
ACS	27 (18.0%)
LVF	21 (14.0%)
PCI	20 (14.0%)
CABG	81 (55.0%)
Arrhythmia	1 (0.7%)
Other cardiac events	7 (4.7%)
Stroke	1 (0.7%)
Bleeding	4 (2.7%)
Other non-cardiac	16 (11.0%)
Unknown	1 (0.7%)

Summary statistics presented as n (%).

*CATH* Catheterization, *ACS* Acute Coronary Syndrome, *LVEF* Left Ventricular Ejection Fraction, *MI* Myocardial Infarction, *PCI* Percutaneous Coronary Intervention, *CABG* Coronary Artery Bypass Graft, *CVA* Cerebrovascular Accident, *PCI* Percutaneous Coronary Intervention, *RBC* Red Blood Cells, *LVF* Left Ventricular Failure.

*n = total number of adverse events; n_i_ = individual adverse event.

### Data processing

We used R software environmental and multiple R statistical packages for all the subsequent statistical analyses. We used a multi-algorithm ML ensemble statistical framework^8,23^, that constructs predictive models by comparing the performance of five supervised algorithms, including random forest (RF), gradient boosting (GB), extreme gradient boosting (XGB), support vector machine (SVM), and logistic regression (LR). Features were included in the models using their original forms (i.e., continuous variables were not converted into a different form and included as they are). We reduced the data dimensionality by excluding features with the largest mean absolute correlation (ρ > 0.9). Then, we used the ‘Boruta’ R package to control for feature multicollinearity by reducing the features to a final set of variables relevant to the subsequent prediction to help improve the performance of the ML models^24^. We used a down-sampling strategy to control for class imbalance which may bias the predictive performance of the algorithms toward the majority class (i.e., patients with no adverse events)^25^. Briefly, this strategy down-samples the majority class to match its frequency with the minority class (i.e., patients with adverse events). For example, for the in-hospital model, we randomly downsampled the majority class by a factor of 5 (i.e., 269 patients with adverse events to 341 patients with no adverse events). However, for the 30 day model we down sampled the major class by a factor of 10 (see Supplementary File 1 & 2) At the same time, we randomly partitioned the data into training (80%) and testing (20%) testing sets using the K-fold cross-validation (CV) method (K = 10) to train and evaluate the ML models. Using the K-fold CV approach, we further split our data into 10 subsets (or folds) and iteratively trained (80% of the fold) and tested (20% of the fold) the model on each fold. Unlike the common cross-validation approach, which divides the data into single training and testing sets, the K-fold CV procedure can guard against overfitting and artificial inflation of the validation parameters described below^26^.

### Model training and evaluation

We trained and created ML predictive models for post-catheterization adverse events using the set of features summarized in Supplementary Table 1. We used the ‘Random Forest’ R package^27^ to run the RF algorithm while we ran the GB, XGB, SVM, and LR using the ‘Caret’ R package^28^. We used the tenfold cross-validation methods to estimate the validation parameters of each algorithm and evaluate their predictive performance. The validation parameters were estimated by averaging the confusion matrix across all of the 10 folds (described above) and included the receiver operator characteristic (ROC), accuracy (Acc), sensitivity (Se), specificity (Sp), and Matthew’s correlation coefficient (MCC). We used the for the training process of all models. The Caret R has been designed to accommodate the Tidyverse R coding structure and workflow (https://www.tidyv erse.org/), which is also the basis for our statistical framework implemented in this study (see Supplementary Files 1 & 2). The Tidymodels coding structure intuitively allows users to utilize a variety of data preprocessing steps, such as data imputation and dealing with imbalanced datasets (as described above). Also, the Caret package (based on the Tidymodels approach) provides a semi-automatic streamlined approach for tuning and optimizing models’ hyperparameters. Because it is difficult to determine priori the exact hyperparameter values^29^, for all of the selected ML algorithms, we used default grid parameter setting in the Tydimodels syntax to train and select the best-performing model automatically. More specifically, we used the “train” function, implemented in the Caret package, which extensively resamples the grid, to evaluate how the selected values of each tuning parameter, such as learning rate, can improve model prediction^30^. A comprehensive guide to the default hyperparameters used in our selected ML algorithms is provided at www.tidymodels.org/find/parsnip/. We then selected the best predictive model for the probability of post-catheterization adverse events by comparing the estimated validation parameters of each algorithm using the testing dataset.

### Model interpretation

We used the best-performing predictive model of each outcome to infer feature importance, partial dependence, interaction strength, and relationships between the features and the adverse events in randomly selected individual patients. Feature importance was computed using Breiman’s method implemented in ‘iml’ R package^31,32^. We then calculated the global and individual effects of each feature on the outcome and each observation from the dataset for the top six important features. We plotted these effects using partial dependence (PD) plots and centered individual conditional expectation (cICE), respectively^33^. We used Friedman’s *H*-statistic to infer feature interaction strength^34^. Briefly, Friedman’s *H*-statistic utilizes the partial dependency decomposition to flexibly quantify feature interaction strength, which represents the proportion of the variance in the data explained by the interaction^34^. Finally, we computed Shapley values (*φ*) to estimate individual-level risk predictions for randomly selected patients and the contribution of each feature to those predictions^35^, which are based on a game theory approach.

## Results

For the in-hospital adverse events model, the RF algorithm outperformed (AUC = 0.84; Table 2) other algorithms in terms of predictive performance (i.e., AUC, Acc, Se, Sp, MCC; Table 2) and correctly predicted 84% of the events (Acc = 0.81). However, the XGB algorithm outperformed other algorithms (AUC = 0.79; Table 2) in correctly predicting adverse events 30 days after discharge (Acc = 0.78; Table 2). Notably, while the LR model performance was fair for predicting in-hospital adverse events, it had the poorest predictive performance for the 30 days adverse events model (AUC = 0.58; Table 2).

**Table 2:** Comparative cross-validation performance parameters for the machine learning models of each outcome.

Model	AUC ± SE	Accuracy (%) ± SE	Specificity (%) ± SE	Sensitivity (%) ± SE	MCC ± SE
In-hospital adverse events					
RF	0.84 ± 0.16	80.92 ± 1.27	81.29 ± 1.31	66.86 ± 1.56	0.40 ± 0.00
GB	0.71 ± 0.01	71.15 ± 2.16	64.12 ± 2.25	64.12 ± 3.88	0.28 ± 0.00
XGB	0.65 ± 1.01	67.19 ± 1.46	68.67 ± 1.49	67.70 ± 0.49	0.23 ± 0.00
SVM	0.78 ± 0.03	73.83 ± 1.76	73.90 ± 1.82	69.62 ± 1.74	0.37 ± 0.00
LR	0.72 ± 0.40	74.80 ± 2.33	75.97 ± 2.38	66.71 ± 1.55	0.19 ± 0.00
30 Days adverse events					
RF	0.68 ± 0.00	62.24 ± 1.39	62.18 ± 1.42	65.92 ± 1.25	0.33 ± 0.00
GBM	0.77 ± 0.01	68.76 ± 1.69	69.67 ± 1.74	70.66 ± 1.71	0.36 ± 0.00
XGB	0.79 ± 1.06	78.01 ± 1.80	74.06 ± 1.83	71.7 ± 1.98	0.38 ± 0.00
SVM	0.71 ± 0.02	62.55 ± 1.76	62.46 ± 1.82	67.50 ± 1.59	0.34 ± 0.00
LR	0.58 ± 0.10	63.89 ± 2.66	61.79 ± 1.34	58.15 ± 1.88	0.32 ± 0.00

MCC = Matthew’s correlation coefficient. Model highlighted in gray is the best performing model.

*AUC* Area Under the Curve, *SE* Standard Error, *RF* Random Forest, *GM* Gradient Boosting, *XGB* Extreme Gradient Boosting, *SVM* Support Vector Machine, *LR* Logistic Regression.

**Figure 1:**
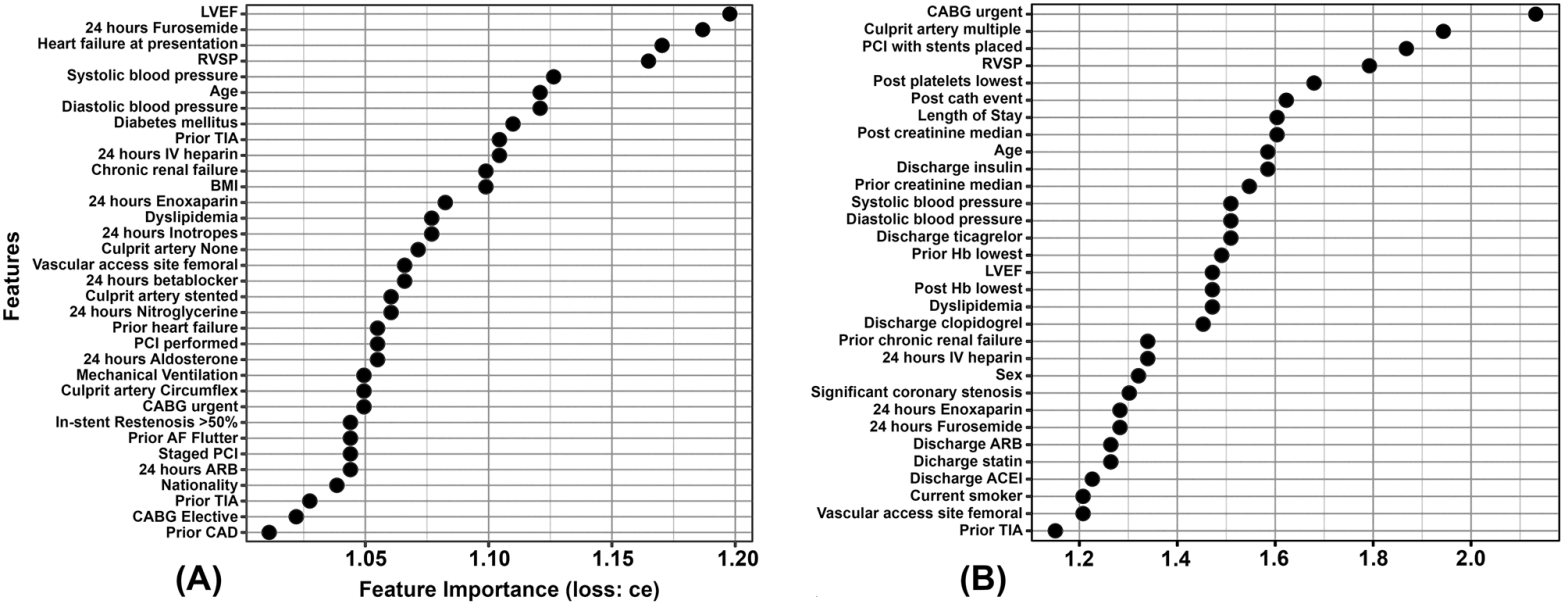
Relative importance of the features in their contribution to the study outcomes. (**A**) In-hospital adverse events. (**B**) 30 days adverse events. ce = classification error loss function (“ce”) used to compute feature importance. Black dots indicate median “ce”. *CATH* Catheterization, *CAD* Coronary Artery Disease, *ACS* Acute Coronary Syndrome, *CCS* Canadian Cardiovascular Society, *LVEF* Left Ventricular Ejection Fraction, *RVSP* Right Ventricular Systolic Pressure, *AF* Atrial Fibrillation, *MI* Myocardial Infarction, *PCI* Percutaneous Coronary Intervention, *CABG* Coronary Artery Bypass Graft, *TIA* Transient Ischemic Attack, *CVA* Cerebrovascular Accident, *STEMI* ST-segment Elevated Myocardial Infarction, *NSTE-ACS* Non ST-segment Elevation Acute Coronary Syndrome, *ACEI* Angiotensin Converting Enzyme Inhibitor, *ARB* Angiotensin Receptor Blocker, Coronary Artery, *CABG* Coronary Artery Bypass Graft, *Hb* Hemoglobin, *BMI* body mass index.

Our ML statistical framework showed that left ventricular ejection fraction (LVEF), followed by furosemide administrated in the first 24 h after catheterization, heart failure, right ventricular systolic pressure (RVSP), systolic blood pressure, and age were the top six important features for predicting in-hospital adverse events (Fig. 1A). Nevertheless, the 30 days adverse events model revealed that urgent coronary artery bypass graft (CABD) followed by the type of culprit artery, percutaneous coronary intervention (PCI) with stents places, RVSP, the post-catheterization platelets lowest concentration, and the occurrence of an in-hospital adverse event were the most important predictors for the 30 days model (Fig. 1B).

**Figure 2:**
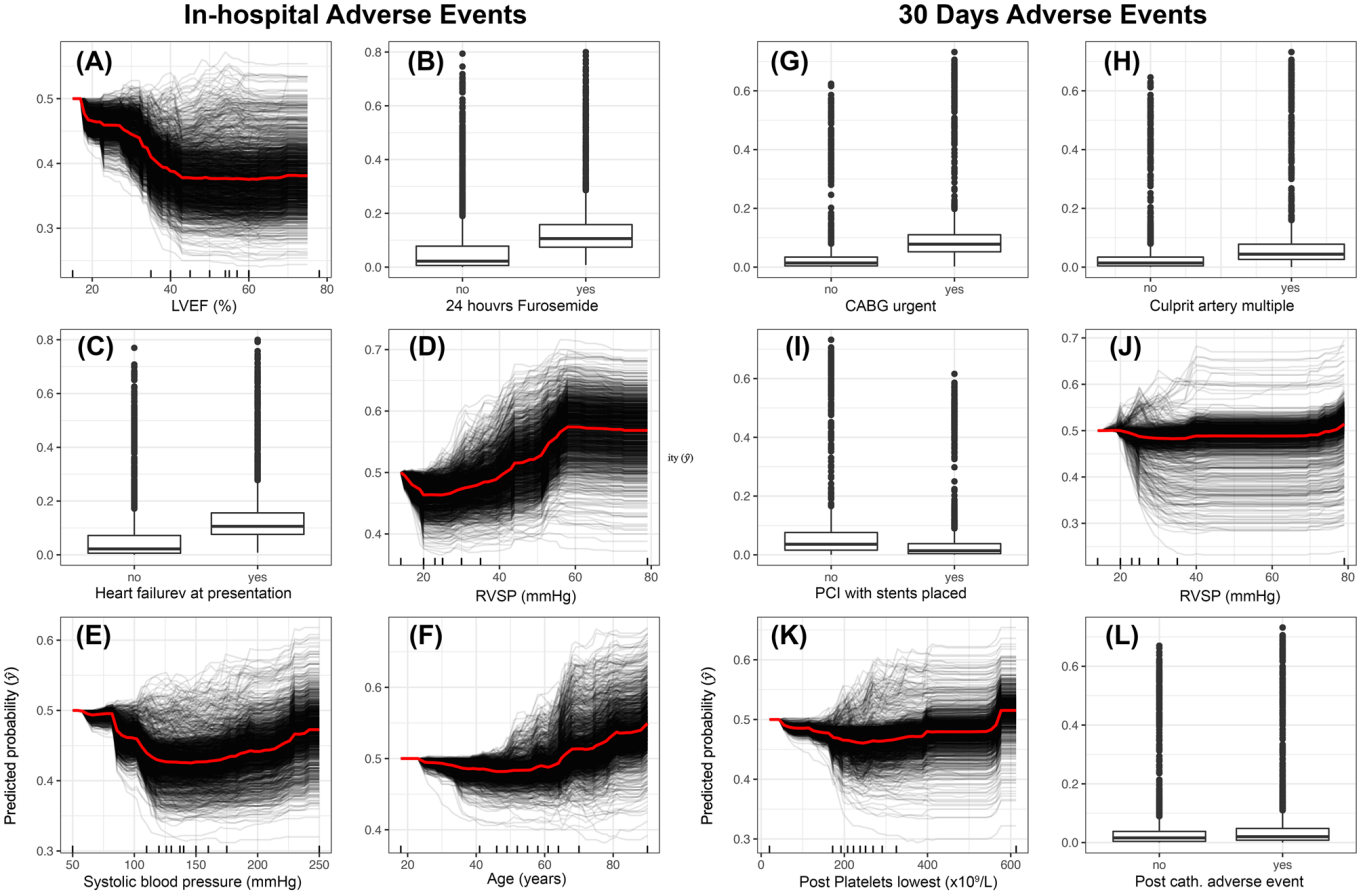
Centered Individual Conditional Expectation (cICE) summary plots for the top six important features that contributed to predicted risk of each outcome. (**A**–**F**) In-hospital adverse events. (**G**–**L**) 30 days adverse events. *CATH* Catheterization, *LVEF* Left Ventricular Ejection Fraction, *RVSP* Right Ventricular Systolic Pressure, *CABG* Coronary Artery Bypass Graft, *PCI* Percutaneous Coronary Intervention.

PD plots revealed that the risk of in-hospital adverse events increased when patients had an approximate LVEF value of less than 40% (Fig. 2A), furosemide in the first 24 h after catheterization (Fig. 2B), heart failure at presentation (Fig. 2C), an RVSP value greater than 40 mmHg (Fig. 2D), systolic blood pressure less than 100 and greater than 200 mmHg (Fig. 2E), and aged above 60 years old (Fig. 2F). However, patients who had urgent CABG (Fig. 2G), multiple culprit arteries (Fig. 2H), PCI with stents placed (Fig. 2I), RVSP value greater than 40 mmHg (Fig. 2J), lowest post catheterization platelets concentrations greater than 400 × 10^9^/L (Fig. 2K) and an ACS post catheterization in-hospital adverse event (Fig. 2L) are more likely to experience a 30 days adverse event after discharge.

**Figure 3:**
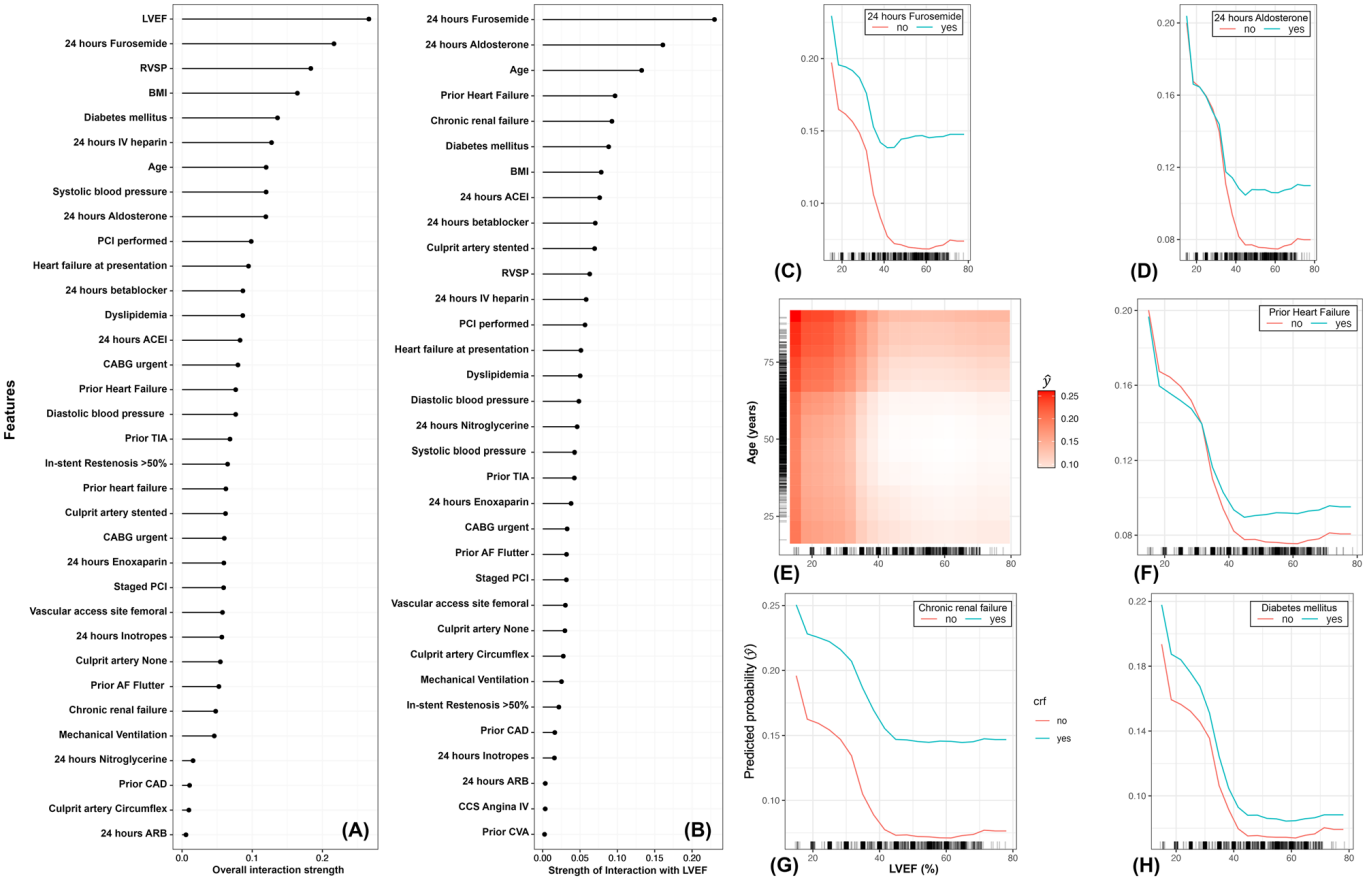
Plots showing important feature interactions that shape the risk of in-hospital adverse events after catheterization are calculated using Friedman’s H-statistic. (**A**) Ranks the overall strength of interaction of each feature with other features; (**B**) Ranks the overall strength of interaction of each feature with % left ventricular ejection fraction (LVEF); (**C**–**H**) Partial dependence plots at the right represent the top six interactions that shaped the risk of in-hospital adverse events. (**C**) interaction between 24 h furosemide and LVEF; (**D**) interaction between 24 h aldosterone and LVEF; (**E**) interaction between Age and LVEF; the heat matrix corresponds to the risk of in-hospital adverse effects, in which lighter shades of red indicate lower risks, and darker shades of reds indicate higher risks; (**F**) interaction between prior history of heart failure and LVEF; (**G**) interaction between chronic renal failure and LVEF; (**H**) interaction between diabetes and LVEF. (**C**–**H**) tick marks on the x-axis show the observed individual values of the patients enrolled in the study. *CAD* Coronary Artery Disease, *ACS* Acute Coronary Syndrome, *CCS* Canadian Cardiovascular Society, *LVEF* Left Ventricular Ejection Fraction, *RVSP* Right Ventricular Systolic Pressure, *AF* Atrial Fibrillation, *MI* Myocardial Infarction, *PCI* Percutaneous Coronary Intervention, *CABG* Coronary Artery Bypass Graft, *TIA* Transient Ischemic Attack, *CVA* Cerebrovascular Accident, *STEMI* ST-segment Elevated Myocardial Infarction, *ACEI* Angiotensin Converting Enzyme Inhibitor, *ARB* Angiotensin Receptor Blocker, Coronary Artery, *Hb* Hemoglobin, *BMI* body mass index.

**Figure 4:**
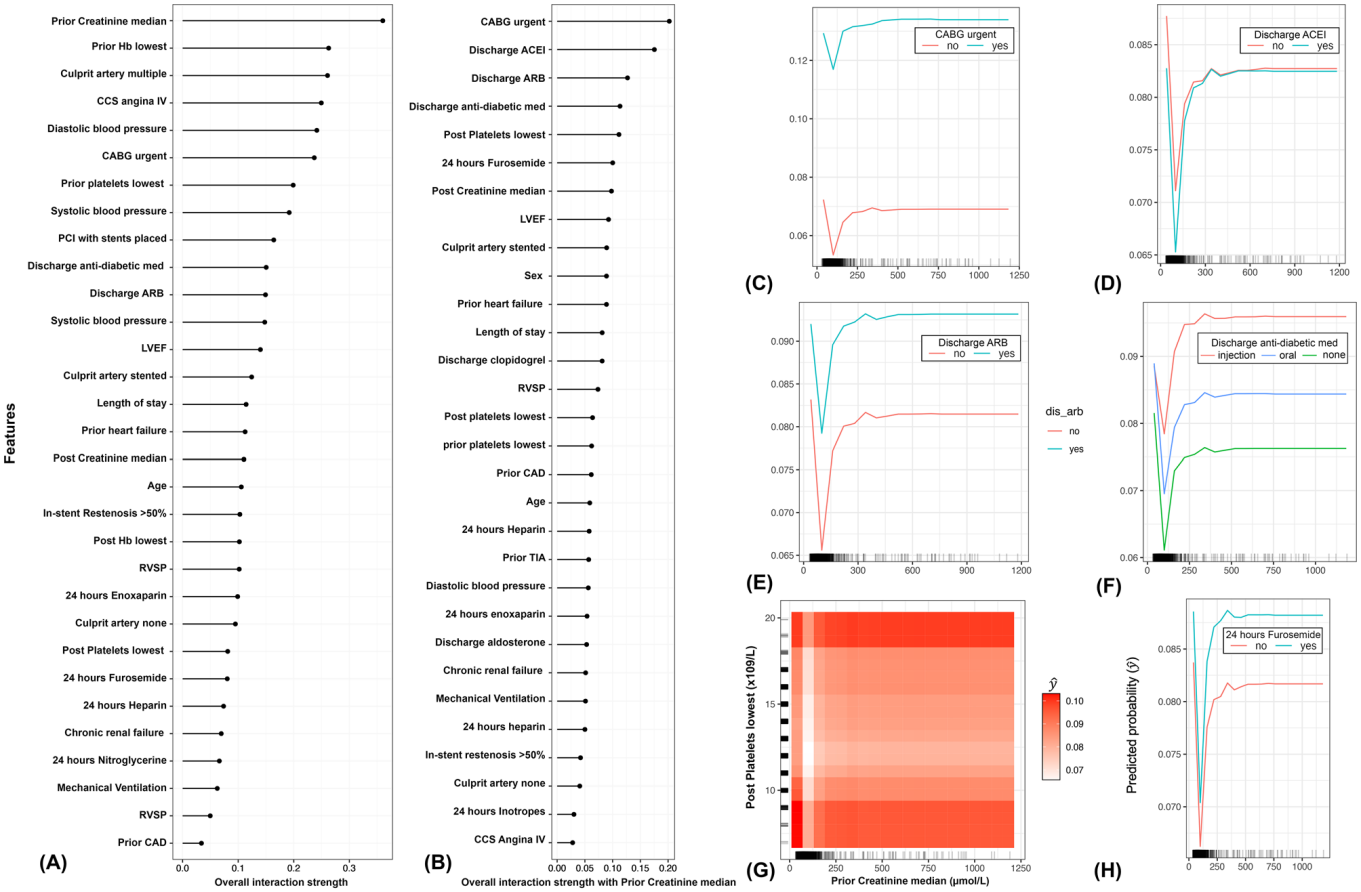
Plots showing important feature interactions that shape the 30-day adverse events after discharge calculated using Friedman’s H-statistic. (**A**) Ranks the overall strength of interaction of each feature with other features; (**B**) Ranks the overall strength of interaction of each feature with prior creatinine median; (**C**–**H**) Partial dependence plots at the right represent the top six interactions that shaped the risk of the 30-days adverse events. (**C**) interaction between urgent CABG and prior creatinine median; (**D**) interaction between discharge ACEI and prior creatinine median; (**E**) interaction between discharge ARB and prior creatinine median; (**F**) interaction between discharge type of antidiabetic medication and prior creatinine median; (**G**) interaction between post platelets lowest and prior creatinine median; the heat matrix corresponds to the risk of 30-days adverse effects, in which lighter shades of red indicate lower risks and darker shades of reds indicate higher risks. (**H**) interaction between 24 h furosemide and prior creatinine median. (**C**–**H**) tick marks on the x-axis show the observed individual values of the patients enrolled in the study. *CATH* Catheterization, *CAD* Coronary Artery Disease, *ACS* Acute Coronary Syndrome, *CCS* Canadian Cardiovascular Society, *LVEF* Left Ventricular Ejection Fraction, *RVSP* Right Ventricular Systolic Pressure, *AF* Atrial Fibrillation, *MI* Myocardial Infarction, *PCI* Percutaneous Coronary Intervention, *CABG* Coronary Artery Bypass Graft, *TIA* Transient Ischemic Attack, *CVA* Cerebrovascular Accident, *STEMI* ST-segment Elevated Myocardial Infarction, *NSTE-ACS* Non ST-segment Elevation Acute Coronary Syndrome, *ACEI* Angiotensin Converting Enzyme Inhibitor, *ARB* Angiotensin Receptor Blocker, *LAD* Left Anterior Descending, *LCX* Left Circumflex, *RCA* Right Coronary Artery, *LIMA* Left Internal Mammary Artery, *RIMA* Right Internal Mammary Artery, *Hb* = Hemoglobin.

We inferred that LVEF on admission had the strongest overall interactions with other features in shaping the risk of in-hospital post-catheterization adverse events (Fig. 3A). Also, we found that medications administrated in the first 24 h after catheterization, such as furosemide (Fig. 3B,C) and aldosterone (Fig. 3B,C) were the top two interacting predictors with LVEF. This is followed by age, in which patients above 60 years old with LVEF values below 40% aggravate the risk of in-hospital adverse events (Fig. 3E). Additionally, our in-hospital adverse events model showed that interactions between patients’ prior history of heart failure, chronic renal failure, and diabetes, on one side and LVEF on other, are significant in increasing the risk of in-hospital adverse events (Fig. 3F–H). Nevertheless, the 30-days adverse events model indicated that the prior median creatinine concentration has the strongest overall interactions with other features (Fig. 4A). Top six most important interacting features are illustrated in Fig. 4B. The model shows that patients undergoing urgent CABG with a prior median blood creatinine concentration greater than 100 µmol/L slightly elevated their risk of 30-day adverse events (Fig. 4C). While the interaction between creatinine concentration and receiving angiotensin-converting enzyme inhibitor (ACEI) on discharge was important (Fig. 4D), the difference in the risk of adverse events for patients receiving it and not receiving was inconclusive (i.e., had no distinct trends). However, patients with prior creatinine greater than 100 µmol/L and on angiotensin receptor blocker (ARB) and insulin injection were more likely to experience an adverse event 30 days after discharge (Fig. 4E,F). Further, our model captured a significant interaction between prior creatinine and post-catheterization lowest platelet concentrations (Fig. 4G). Here, we found that the risk is remarkably increased at prior creatinine greater than 100 µmol/L with either platelet concentrations above 18 × 10^9^/L and less than 8 × 10^9^/L. Also, like the in-hospital adverse events model, the 30-days model suggests significant interaction between creatinine concentration and receiving furosemide within 24 h after catheterization (Fig. 4H).

**Figure 5:**
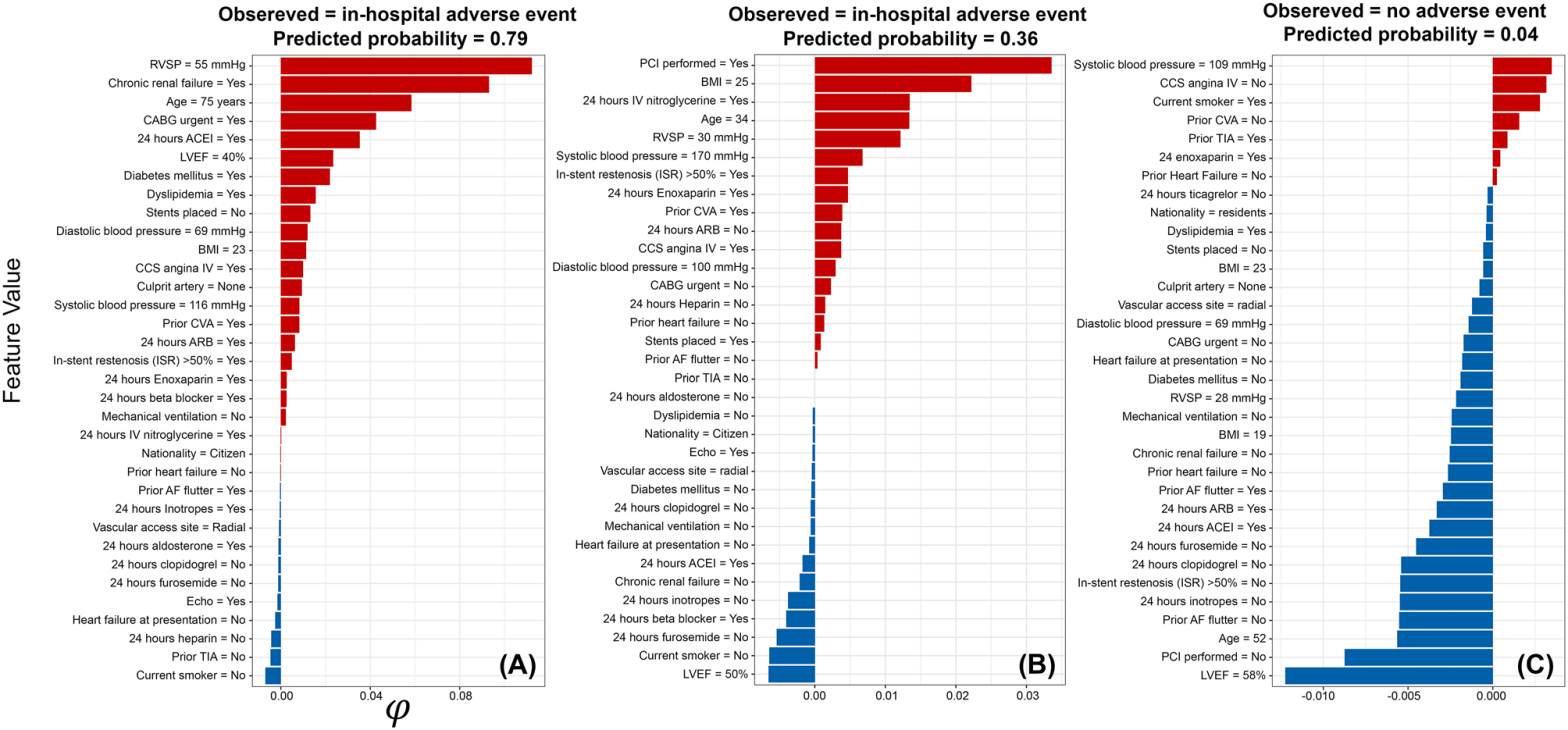
Feature value contributions for the respective risk of in-hospital adverse events based on Shapley Values (φ) for three individual patients. (**A**) a patient who had multiple severe in-hospital adverse events; (**B**) a patient who had moderate in-hospital adverse events; (**C**) a patient who did not have an in-hospital adverse event after catheterization; Red bars represent positive Shapley values and indicate that this feature increased the risk of the outcome, while blue bars represent negative Shapley values and suggest that this feature decreased the risk of the outcome. The values next to each feature represent the observed value of that feature for that patient. *CCS* Canadian Cardiovascular Society, *LVEF* Left Ventricular Ejection Fraction, *RVSP* Right Ventricular Systolic Pressure, *AF* Atrial Fibrillation, *MI* Myocardial Infarction, *PCI* Percutaneous Coronary Intervention, *TIA* Transient Ischemic Attack, *CVA* Cerebrovascular Accident, *STEMI* ST-segment Elevated Myocardial Infarction, *NSTE-ACS* Non ST-segment Elevation Acute Coronary Syndrome, *ACEI* Angiotensin Converting Enzyme Inhibitor, *ARB* Angiotensin Receptor Blocker, *CABG* Coronary Artery Bypass Graft, *Hb* Hemoglobin, *BMI* body mass index.

**Figure 6:**
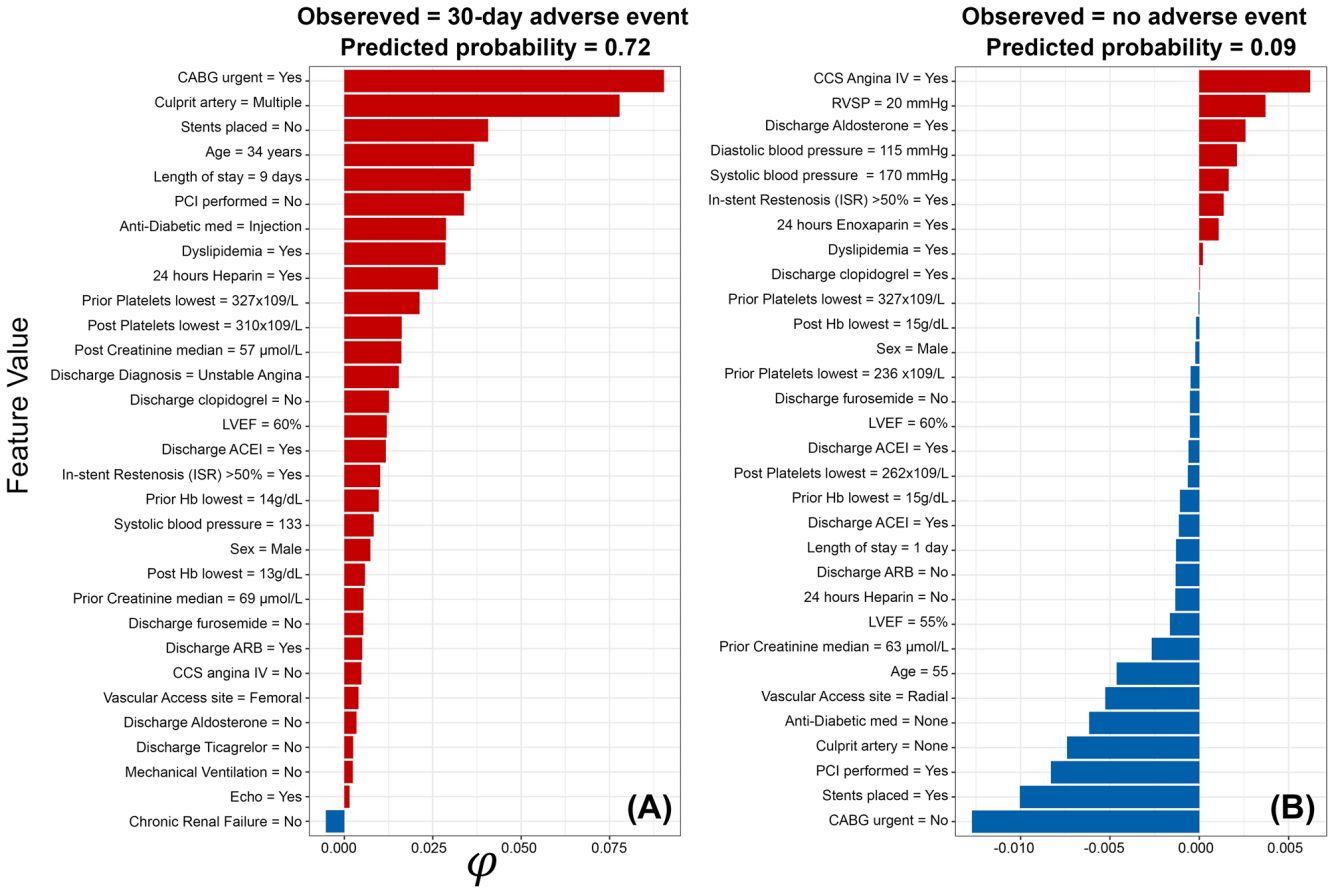
Feature value contributions for the respective risk of 30-days adverse events based on Shapley Values (φ) for two individual patients. (**A**) a patient who had an adverse event 30 days after discharge; (**B**) a patient who did not have an adverse event 30 days after discharge. Red bars represent positive Shapley values and indicate that this feature increased the risk of the outcome, while blue bars represent negative Shapley values and suggest that this feature decreased the risk of the outcome. The values next to each feature represent the observed value of that feature for that patient. *CCS* Canadian Cardiovascular Society, *LVEF* Left Ventricular Ejection Fraction, *RVSP* Right Ventricular Systolic Pressure, *AF* Atrial Fibrillation, *MI* Myocardial Infarction, *PCI* Percutaneous Coronary Intervention, *TIA* Transient Ischemic Attack, *CVA* Cerebrovascular Accident, *STEMI* ST-segment Elevated Myocardial Infarction, *NSTE-ACS* Non ST-segment Elevation Acute Coronary Syndrome, *ACEI* Angiotensin Converting Enzyme Inhibitor, *ARB* Angiotensin Receptor Blocker, *CABG* Coronary Artery Bypass Graft, *Hb* Hemoglobin, *BMI* body mass index.

Our Shapley value estimates by our final models suggest that a patient is more likely to experience several in-hospital adverse events simultaneously, including contrast-induced nephropathy, heart failure, arterial fibrillation, new requirement for dialysis, and RBC whole blood transfusion (probability = 0.79), when their RVSP is equal to 55 mmHg, with chronic renal failure, aged 75 (Fig. 5A). While a patient observed with in-hospital acute thrombosis (probability = 0.28), was characterized by the RVSP is equal to 35 mmHg, with prior CVA and PCI performed (Fig. 5B). In contrast, in an observed patient with LVEF equal to 58% at presentation, no PCI was performed, aged 52 is less likely to experience in-hospital adverse events (probability = 0.04) after catheterization (Fig. 5C). Also, we inferred that patient had urgent CABG with multiple culprit arteries and stent placed are most likely to be re-hospitalized 30 days after discharge, due to reoccurring ACS event, that require another CABG, (probability = 0.72; Fig. 6A). However, if an observed patient had no need for urgent CABG, but performed a PCI with stents placed is less likely to experience adverse events after discharge (probability = 0.09; Fig. 6B).

## Discussion

We used an interpretable ML statistical framework and the Kuwait CLAP registry data to reveal deeper insights into the risk factors that shape the outcomes of admitted patients with ACS during hospital stay and at 30 days from discharge. Also, we demonstrated how our ML analytical pipeline could untangle the unique and complex relationships between the different risk factors related to patient characteristics and in-hospital clinical procedures. Also, we showed that our most important predicting features had remarkable non-linear relationships with other baseline characteristics in shaping the risk of clinical outcomes. These findings not only support and improve clinical practice but assist with alleviating the public health and economic implications of ACSs.

Our in-hospital adverse events ML model identified LVEF as the most important risk factor (Fig. 1) with the highest interaction strength with other features (Fig. 3). This is unsurprising since many past studies highlighted the critical role of low LVEF values in influencing the risk of post-catheterization in-hospital adverse events^36–39^. Here, our cICE plot demonstrated that LVEF values on the admission of less than 40% increase the risk of post-catheterization in-hospital events (Fig. 2A). However, this risk is aggravated particularly in older patients (Figs. 2F and 3E) with heart failure (Figs. 2C and 3F), high RVSP (Fig. 2D) and irregular systolic blood pressure (Fig. 2E). All these features represent severe cardiac insufficiency leading to poor long-term prognosis, and thus, need to be taken into consideration when performing any catheterization procedure in ACS patients^39^. Further, our results showed that patients receiving furosemide within 24 h of admission are at elevated risk of in-hospital adverse events (Fig. 2B).

Also, interaction plots illustrated a significant non-linear relationship between low LVEF values and receiving furosemide in amplifying the risk of adverse events (Fig. 3C)^40^. Furosemide is commonly used as a loop diuretic for patients with heart failure. Therefore, our results reflect that patients with severe cardiac outcomes (such as low LVEF) requiring a high dose of furosemide may have a poor prognosis^41^. The same may also be implied in patients receiving high doses of aldosterone within 24 h of their admission, as shown in Fig. 3D. Moreover, adverse events may also result from the rare side effect of these medications, thus combining them with other medications may improve their therapeutic outcomes^42^. Additionally, our model not only demonstrated that patients with chronic renal failure and diabetes are at high risk of adverse events as suggested elsewhere^38,43–45^, but revealed significant interactions with low LVEF values (Fig. 3G,H). These findings agree with the notion that the combination of hyperglycemia and renal insufficiency associated with low LVEF values is the leading cause of in-hospital adverse events, particularly in patients who have undergone a PCI operation^38^. Also, these poor outcomes might reflect the low cardiac output, hemodynamic instability, and reduced renal blood flow, which leads to hypoxia and the generation of reactive oxygen species^46^.

Nevertheless, the 30-days adverse event model inferred that highly invasive intervention procedures, such as performing urgent CABG, having multiple culprit arteries, with stents placed during PCI, are significant predictors of poor outcomes after discharge (Fig. 2G–I). These findings confirm the results of past studies in terms of reflecting the severity of the patient's ACS condition^16,47^. Also, this is evidenced by the importance of the cardiological and hematological indicators such as RVSP and platelets, respectively (Fig. 2J,K), as well as having an in-hospital adverse event (Fig. 2L). However, unlike previous inferences^3,6,47^, our model uncovered the strong non-linear relationships between admission creatinine levels (Fig. 4A) and other features (Fig. 4B) in shaping the risk of adverse events after discharge (Fig. 4A). Here, our inferences demonstrate that patients requiring urgent CABG with creatinine levels less than 50 µmol/L or greater than 100 µmol/L are more likely to experience a poor post-operative prognosis (Fig. 3C). This result is expected since abnormal serum creatinine levels correspond to other comorbidities, particularly chronic kidney disease, exacerbating the long-term risk of postoperative adverse events^48^. Similarly, serum creatinine had a strong non-linear relationship with ACEI and ARB intake after discharge (Fig. 4D,E) in hypertensive patients.

Nonetheless, our results show minor discrimination in the risk between patients discharged with and without ACEI (Fig. 4D). In contrast, remarkable discrimination was inferred between patients discharged with and without ARB medication (Fig. 4E). These findings quantify the notion that ARB may increase the risk of myocardial infarction (MI) in hypertensive patients, and therefore, dispensing ACEI to control their blood pressure may be more appropriate, particularly for acute MI patients, as suggested elsewhere^49^. Also, our model was able to discriminate the broad spectrum of risk of poor outcomes among diabetic patients with abnormal serum creatinine levels (Fig. 4F). These results suggest that severely diabetic patients (i.e., who are under insulin injection as a proxy) are more likely to experience adverse events than moderately diabetic patients (i.e., who are under oral medication). Indeed, the complex angiographic pattern extending between the mid and distal arteries of ACS patients with severe diabetes is characterized by a multivessel diffuse plaque, making revascularization quite challenging for clinicians^50^. Thus, interventional cardiologists and cardiothoracic surgeons might need to implement an individualized approach with a multidisciplinary heart team on severely diabetic patients to minimize poor outcomes after discharge^50^.

One limitation of this study is the aggregation of positive outcomes into one category in our cohort. Yet, the rarity, complexity and broad spectrum of outcomes (Table 1) made it difficult for us to generate a representative model for each adverse event. However, the aggregation of the adverse events increased our computational efficiency, substantially improved the predictive performance of our ML algorithms, and facilitated the practical interpretation of our models. A second limitation of the Kuwait CLAP registry is the population size, and therefore generalizability of our inferences might be biased toward the population that comprised our analyses. That said, many of our findings agree with past studies regarding short- and long-term adverse events resulting from post-catheterization. Furthermore, our analysis mainly focuses on revealing complex relationships in the available data that might be useful for improving clinical decision-making related to the diagnostic and prognostic efforts in the same population where the data were retrieved. This is in addition to the fact that data is being collected from only sites that provide cardiological services in the country, as described above, making it representative of the whole population of Kuwait. Also, our k-fold cross-validation procedure lessens the chances of overfitting, increasing the robustness of its subsequent inferences. Nevertheless, future studies will be aimed at applying our analytical pipeline on a larger sample size and will be focused on building specific models for the most prevalent adverse events.

The complexity of ACSs epidemiology, the growing volume of cardiac intervention procedures with their related data, and the highly non-linear relationships between patient baseline characteristics, clinical procedures, and interventions highlight the utility and robustness of our ML statistical framework. One important highlight of our analytical pipeline is the ability to flexibly explore heterogeneous treatment effects (i.e., effect modification and beyond) comprising multiple features simultaneously rather than overall average intervention effects using one-way or more interaction terms as in traditional regression models^51^. Due to the tedious task of modelling and interpreting all possible interaction terms, rigorous evaluation of heterogeneous treatment effects has yet to be widely explored in clinical epidemiology^52^. As shown in Figs. 3 and 4, investigators can intuitively interrogate multiple interactions to capture clusters of subgroups showing different feature-outcome effects. For example, Fig. 3G simultaneously shows how the risk of adverse in-hospital events has distinct patterns of over six significant interactions. In these interactions, the highest risk of adverse events notably peaks over certain clusters of patients with specific interrelated features (Fig. 3C–H). This allows clinicians to assess the effectiveness of their interventions and formulation of targeted approaches for reducing cardiovascular adverse events for individual clusters of patients. Wiemken and Kelley., 2020 extensively discussed the advantage of the ML algorithmic approach in dealing with interactions, as well as how traditional stratified regression models and the inclusion of interaction terms can lead epidemiologists to the issue of multiple testing bias^51^.

Here, our ML models had good and similar predictive performance compared to past studies in terms of evaluation parameters (e.g., AUCs = 0.84 & 0.79 for the in-hospital and 30-day adverse events models, respectively, Table 2)^7,14,53–55^. Further, we showed how RF and XGM algorithms can remarkably outperform traditional models such as logistic regression (Table 2). Subsequently, many studies also demonstrated that our statistical approach outperforms standard risk stratification tools such as TIMI and GRACE^7,17,54^. However, many of these ML studies mainly focused on their models' predictive power (i.e., using a black-box approach), which they did not embrace their interpretability in a clinical setting. Hence, a readily interpretable model will provide new insights into the complex epidemiology of adverse events and be easily adopted by cardiologists to be implemented in their practice. Given that the Middle East has the highest incidence of CADs on a global scale^4^, our study represents the first attempt to utilize an interpretable ML statistical framework focused on uncovering complex relationships to improve clinicians’ intervention efforts.

Besides the inherent limitations of the statistical framework used to build standard risk stratification tools, the generalizability of their inferences might also be restricted to specific populations. Indeed, the environmental, genetic, and clinical settings and resources might differ substantially between countries and regions worldwide. Thus, a customized risk stratification tool based on local data will provide more plausible and generalizable inferences for its source population than global-based tools. Therefore, we further elucidate the remarkable applicability of Shapley values, a game theoretic approximation, to interrogate in finer scales what each model represents regarding the predicted risk of adverse events (e.g., why a particular patient had a poor post-catheterization outcome, while the other did not?). For example, the in-hospital model inferred remarkably different magnitudes of risk for different types of adverse events in individual patients instead of averaging over the risk profiles of these patients (Fig. 5). Here, our model predicted high probabilities for specific adverse events (P = 0.79; Fig. 5A), such as in-hospital heart failure and contrast-induced nephropathy in older patients with chronic renal failure who had an urgent CABG. However, midrange probabilities were predicted for other adverse events, such as acute thrombosis (P = 0.28; Fig. 5B), in younger patients with prior CVA and who had a basic PCI. Thus, both types of patients had notably distinct demographics and clinical features with different requirements for in-hospital procedures. Additionally, for a randomly selected patient who had an adverse event 30 days after discharge, having an urgent CABG with multiple culprit arteries and stents placed during PCI put that patient at high risk of having a poor outcome (i.e., 72% chance; Fig. 6A). In contrast, under the same predictive model, the other selected patient who had no adverse events 30 days after discharge, entirely lacks such risk profile (Fig. 6B).

Finally, the Shapely statistical procedure assigns positive and negative values for the features that increased and/or decreased the probability of adverse events in individual patients, respectively (Figs. 5 and 6). Hence, using such an intuitive approach can provide additional guidance to the clinician’s diagnostic and prognostic efforts and aid in allocating intervention resources to patients at higher risk, whether in-hospital or after discharge. Yet, additional evaluation of the technical feasibility and clinical plausibility are crucial steps before integrating such predictive models into the standard healthcare systems^15^.

## Conclusion

The incidence of ACS has startlingly doubled over the past few years, and thus, the unparalleled rising demand for cardiological interventions is increasingly prompting healthcare professionals to seek novel methods of anticipating adverse events and accordingly better allocate their limited resources to enhance patient outcomes and decrease long-term public health and economic implications. Further applications of our interpretable ML statistical framework to guide interventions will help improve the quality of life for both health professionals and their patients. In this study, we generally found that presenting symptoms on admission and catheterization procedures were the important variables shaping the risk of in-hospital and 30-day adverse events, respectively. While worth noting that these two sets of features are considered proxies for the severity of the patient's condition. We illustrated how our models outperformed traditional statistical and risk stratification methods due to their minimal statistical assumptions, ability to quantify complex non-linear relationships and elucidate individual patient-predicted risk based on their unique characteristics in finer scales. To our knowledge, fully interpretable ML models have not been widely used in the Middle East. Thus, our ML-based risk stratification approach can improve clinicians’ intervention efforts by providing precise epidemiological insights into ACS adverse events.

### Supplementary Information

Supplementary Table S1.

Supplementary Information 1.

Supplementary Information 2.

Supplementary Legends.

## Data Availability

All of the data relevant to this study were summarized in the body of the manuscript, figures and tables. Original raw data can be provided upon a reasonable request by the corresponding author Moh A. Alkhamis (m.alkhamis@ku.edu.kw).
