# Supplementary material for: Interpretable machine learning models for predicting in-hospital and 30 days adverse events in acute coronary syndrome patients in Kuwait
Source: Sci Rep. 2024 Jan 12;14:1243. doi: 10.1038/s41598-024-51604-8 (PMC10786865; doi:10.1038/s41598-024-51604-8)
Supplement: Supplementary file 1 — Supplementary Table S1. [file 41598_2024_51604_MOESM1_ESM.docx]

**Interpretable Machine Learning Models for Predicting** **In-hospital and 30 Days Adverse Events in Acute Coronary Syndrome Patients in Kuwait**

Moh A. Alkhamis^1^, Mohammad Al Jarallah, Sreeja Attur, Mohammad Zubaid

**Supplementary Table 1. Baseline characteristics of the study population.** Summary statistics presented as either n (%) or mean ± standard deviation (SD). CATH = Catheterization, CAD = Coronary Artery Disease, ACS = Acute Coronary Syndrome, CCS = Canadian Cardiovascular Society, LVEF = Left Ventricular Ejection Fraction, RVSP = Right Ventricular Systolic Pressure, AF = Atrial Fibrillation, MI = Myocardial Infarction, PCI = Percutaneous Coronary Intervention, CABG = Coronary Artery Bypass Graft, TIA = Transient Ischemic Attack, CVA = Cerebrovascular Accident, STEMI = ST-segment Elevated Myocardial Infarction, NSTE-ACS = Non ST-segment Elevation Acute Coronary Syndrome, ACEI= Angiotensin Converting Enzyme Inhibitor, ARB= Angiotensin Receptor Blocker, LAD = Left Anterior Descending, LCX = Left Circumflex, RCA = Right Coronary Artery, LIMA = Left Internal Mammary Artery, RIMA = Right Internal Mammary Artery, Hb= Hemoglobin.

| **Characteristic** | **n = 1976** |
| --- | --- |
| *Demographic Characteristics* | |
| Age (years) | 55.0 ± 11.0 |
| Sex (male) | 1695 (85.0%) |
| Nationality (residents) | 1498 (75.0%) |
| BMI (kg/m2) | 20.0 ± 3.3 |
| Current smoker | 869 (44.0%) |
| *Past Medical History* | |
| Hypertension | 1090 (55.0%) |
| Family history premature CAD | 262 (13.0%) |
| Dyslipidemia | 810 (41.0%) |
| Diabetes Mellitus | 907 (46.0%) |
| Chronic Renal Failure | 130 (6.6%) |
| Prior Heart Failure | 118 (5.9%) |
| Prior AF Flutter | 68 (3.4%) |
| Prior CAD  Prior MI  Prior coronary artery stenosis >50%  Prior Angina  Prior PCI  Prior CABG | 570 (29.0%)  389/570 (68.0%)  352/389 (90.0%)  442/570 (78.0%)  420/570 (74.0%)  86/570 (15.0%) |
| Prior TIA | 24 (1.2%) |
| Prior CVA | 53 (2.7%) |
| *Presenting Symptoms on Admission* | |
| CCS Angina IV | 850 (43.0%) |
| Heart failure at time of presentation | 198 (10.0%) |
| Mechanical Ventilation | 41 (2.1%) |
| Systolic blood pressure (mmHg) | 140 ± 26 |
| Diastolic blood pressure (mmHg) | 83 ± 16.0 |
| Heart (rate/min) | 83 ± 19.0 |
| Echo | 1891 (96.0%) |
| LVEF (%) | 50 ± 12.0 |
| RVSP (mmHg) | 27 ± 8.0 |
| *Medications Administered in First 24 Hours* | |
| Aspirin | 1957 (99%) |
| Clopidogrel loading dose | 1818 (92%) |
| Ticagrelor | 126 (6.4%) |
| IV Unfractionated Heparin | 451 (22.8%) |
| Enoxaparin | 1368 (69.2%) |
| Inotropes | 38 (2.0%) |
| IV Nitroglycerine Tridil | 381 (19.0%) |
| Beta Blocker Medication | 1812 (92.0%) |
| ACEI | 1548 (78.0%) |
| ARB | 162 (8.2%) |
| Aldosterone Blocking Agent | 156 (7.9%) |
| Statin | 1930 (98.0%) |
| Non-statin lipid-lowering Agent Medication | 72 (3.6%) |
| Furosemide IV or Oral | 274 (14.0%) |
| *In-hospital Cath Lab Procedures* | |
| Vascular Access site  Radial  Femoral  Brachial  Other | 1,774 (90.0%)  199 (10.0%)  2 (0.1%)  1 (0.1%) |
| Significant Coronary Stenosis  Left Main  LAD or tributaries  LCX or tributaries  RCA or tributaries  Ramus  Venous grafts  LIMA  RIMA | 1,622 (82.0%)  178/1,622 (11.0%)  1,201/1,622 (74.0%)  859/1,622 (53.0%)  876/1,622 (54.0%)  60/1,622 (3.7.0%)  43/1,622 (2.7.0%)  12/1,622 (0.7%)  2/1,622 (0.1%) |
| In-stent Restenosis (ISR) >50% | 156 (7.9%) |
| Culprit artery  Left Main  Venous Graft  LAD or tributaries  LIMA  Circumflex or tributaries  RIMA  RCA or tributaries  Ramus  None  Multiple | 18 (0.9%)  8 (0.4%)  564 (29.0%)  3 (0.2%)  189 (9.6%)  0 (0.0%)  293 (15.0%)  11 (0.6%)  496 (25.0%)  394 (20.0%) |
| PCI performed  GP IIb/IIIa Inhibitor  Culprit artery stented  Other Vessel stented  Stents placed  Lesion assessment device  Is the patient planned for Staged PCI | 1,219 (61.7%)  196/1,219 (16.0%)  1,136/1,219 (93.0%)  207/1,219 (17.0%)  1,193/1,219 (98.0%)  50/1,219 (4.1.0%)  160/1,219 (13.0%) |
| Urgency of the planned CABG  Elective  Urgent  Emergency  Salvage  CABG was planned during index hospitalization  CABG was planned as an outpatient | 292 (14.7%)  180/292 (62.0%)  104/292 (36.0%)  8/292 (2.7%)  0/292 (0.0%)  137/292 (47.0%)  139/292 (48.0%) |
| *Laboratory Values Prior and Post In-hospital Procedure* | |
| Prior Creatinine median (µmol/L) | 101.0 ± 100.1 |
| Post Creatinine median (µmol/L) | 106.1 ± 104.9 |
| Prior Hb lowest (g/dL) | 14.3 ± 1.9 |
| Post Hb lowest (g/dL) | 13.9 ± 1.8 |
| Prior Platelets lowest (x10^9^/L) | 248 ± 71.1 |
| Post Platelets lowest (x10^9^/L) | 237 ± 65.1 |
| **Discharge Characteristics* | |
| Discharge Diagnosis  STEMI  NSTEMI  Unstable Angina  Stable Angina  Not CAD | 671 (34.0%)  800 (40.0%)  235 (12.0%)  175 (8.7%)  122 (6.1%) |
| Discharge Status  Alive  Dead | 1951 (99.0%)  25 (1.3%) |
| Length of stay (days) | 3 ± 4.0 |
| Discharge medications  Aspirin  Clopidogrel  Ticagrelor  Oral Anticoagulants  Beta Blocker  ACEI  ARB  Aldosterone Blocking Agent  Statin  Non statin lipid lowering Agent  Furosemide  Anti-Diabetic medications  Insulin  Oral treatment | 1857 (94.0%)  1463 (74.0%)  178 (9.0%)  115 (5.8%)  1823 (92.0%)  1476 (75.0%)  177 (8.9%)  248 (13.0%)  1927 (97.0%)  174 (8.8%)  298 (15.0%)  967 (49.0%)  490/967 (51.0%)  606/967 (63.0%) |

*Excluded from the in-hospital adverse event model.
