# Supplementary material for: Interpretable machine learning models for predicting in-hospital and 30 days adverse events in acute coronary syndrome patients in Kuwait
Source: Sci Rep. 2024 Jan 12;14:1243. doi: 10.1038/s41598-024-51604-8 (PMC10786865; doi:10.1038/s41598-024-51604-8)
Supplement: Supplementary file 4 — Supplementary Legends. [file 41598_2024_51604_MOESM4_ESM.docx]

**Supplementary Legends**

Supplementary Table 1. Baseline characteristics of the study population.

Supplementary File 1. R-codes for the in-hospital adverse events ML model

Supplementary File 3. R-codes for the 30-days adverse events ML model
